# Dataset of coded handwriting features for use in statistical modelling

**DOI:** 10.1016/j.dib.2017.12.014

**Published:** 2017-12-13

**Authors:** Anna Agius, Marie Morelato, Sébastien Moret, Scott Chadwick, Kylie Jones, Rochelle Epple, James Brown, Claude Roux

**Affiliations:** a University of Technology Sydney, Centre for Forensic Science, PO Box 123, Broadway 2007, Australia; b Forensics, Specialist Operations, Australian Federal Police, G.P.O. Box 401, Canberra, ACT, Australia; c University of Technology Sydney, School of Mathematical & Physical Sciences, PO Box 123, Broadway 2007, Australia

## Abstract

The data presented here is related to the article titled, “Using handwriting to infer a writer's country of origin for forensic intelligence purposes” (Agius et al., 2017) [1]. This article reports original writer, spatial and construction characteristic data for thirty-seven English Australian^1^ writers and thirty-seven Vietnamese writers. All of these characteristics were coded and recorded in Microsoft Excel 2013 (version 15.31). The construction characteristics coded were only extracted from seven characters, which were: ‘g’, ‘h’, ‘th’, ‘M’, ‘0’, ‘7’ and ‘9’. The coded format of the writer, spatial and construction characteristics is made available in this Data in Brief in order to allow others to perform statistical analyses and modelling to investigate whether there is a relationship between the handwriting features and the nationality of the writer, and whether the two nationalities can be differentiated. Furthermore, to employ mathematical techniques that are capable of characterising the extracted features from each participant.

**Specifications Table**

**Table:** 

**Subject area**	Forensic science
**More specific subject area**	Handwriting examination, forensic intelligence
**Type of data**	Table
**How data was acquired**	Manual coding
**Data format**	Categorical, numerical data
**Experimental factors**	Handwriting specimens were scanned and coded
**Experimental features**	Features of each participants’ handwriting were selected, extracted and coded
**Data source location**	Handwriting specimens were collected from people living in the Sydney region (and whom had learnt to write English in New South Wales). All original documents are stored at the University of Technology Sydney (UTS), 15 Broadway, Ultimo, Sydney, NSW, 2007.
**Data accessibility**	The data is made available with this article
**Related research article**	Agius et al. [1].

**Value of the data**

• This data represents a complementary method for using handwriting features to obtain useful knowledge about the source.
• The data may help to expand the contributions of handwriting examination beyond answering the traditional Court-oriented questions and become more involved in a forensic intelligence framework.
• Researchers may use this data as a building point to trial different statistical techniques and modelling tools in order to determine whether any relationship exists within the dataset.

## 1 Data

The data is presented in Excel spreadsheets, where each column is headed by a particular handwriting characteristic with a description of the relevant codes for each feature. Table 1 below provides a description of the data in each spreadsheet. The first spreadsheet is titled “Writer & Spatial Characteristics”. Writer characteristics are the traits specific to the individual writer and were taken from a survey filled out by each participant (see below). Spatial characteristics are the height and/or width relationships within and between individual characters and words, and how these are combined to form lines of words and paragraphs. The following six spreadsheets (‘h’ and ‘th’ are combined onto one spreadsheet) are the construction characteristics extracted for the characters ‘g’, ‘h’, ‘th’, ‘M’, ‘0’, ‘7’ and ‘9’. Construction characteristics are handwriting features that indicate how the writer forms a letter, number or symbol, e.g. number, position, order and direction of strokes. Please note, Vietnamese writer 11 was not included in the spreadsheets for the construction characteristics of the letters and numbers, as these characteristics could not be extracted from their handwriting due to the writing instrument that they used.

**Table 1:** Summary of the features coded in each of the Excel spreadsheets provided.

**Spreadsheet title**	**Features coded**
Writer & Spatial Characteristics	Writer's ID number; Nationality; Text indented, Valediction indented; Salutation indented; Paragraph 1; Paragraph 2; Slant; Handedness; Number of letters overhanging the right margin; Age; Years they have learnt English; Height of text; Width of text; Area of text; Average height of the letters ‘a’, ‘e’ and ‘o’; and average number of letters per line
Lowercase ‘g’	Writer ID number; Nationality; Handedness; Slant; Allograph; Letter number and the word the letter came from; Letter position within the word; Descending stroke design; Upper loop design; Connected to previous letter; Connected to subsequent letter; Direction of upper loop; Age; and the years they have learnt English
Lowercase ‘h’ and ‘th’	Writer ID number; Nationality; Handedness; Slant; Allograph; Letter number and the word the letter came from; Letter position within word; Connected to previous letter; Connected to subsequent letter; Curve at the top of staff; Relative height of arch to staff; Arch retrace of staff; Arch shape; Height of ‘h’ staff relative to ‘t’ staff; Age; Number of strokes to form ‘th’ combination; and the years they have learnt English
Uppercase ‘M’	Writer ID number; Nationality; Handedness; Slant; Allograph; Letter number and the word the letter came from; Retrace on initial stroke; Apex 1 shape; Apex 2 shape; Apex 3 shape; Relative height of apex 2 to apex 1; Position of apex 2 relative to 1 and 3; Age; Number of strokes; and years they have learnt English
Number ‘0’	Writer ID number; Nationality; Handedness; Slant; Allograph; Number allocated to each ‘0’ written by the participant; Start position; Finished position; Connection to start stroke; Embellishment; Direction; Age; and years they have learnt English
Number ‘7’	Writer ID number; Nationality; Handedness; Slant; Allograph; Number allocated to each ‘7’ written by the participant; Style; Curve at bottom of staff; Angle between the horizontal and vertical strokes; Age; Number of strokes; and years they have learnt English
Number ‘9’	Writer ID number; Nationality; Handedness; Slant; Allograph; number allocated to each ‘9’ written by the participant; Upper loop design; Descending stroke design; Direction of upper loop; Age; Number of strokes; and years they have learnt English

## 2 Experimental design, materials, and methods

**Fig. 1:**
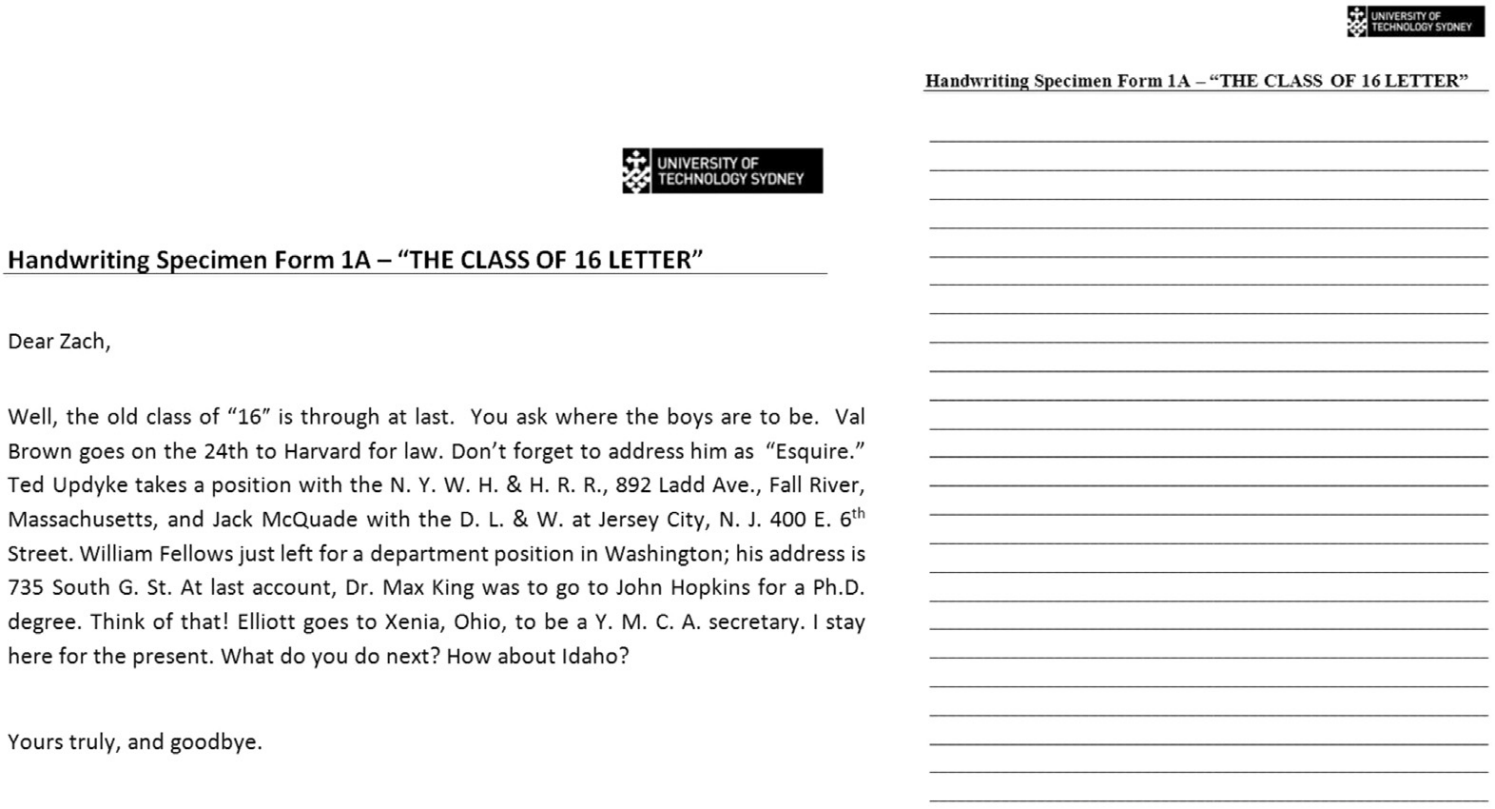
Source document (left) and provided lined piece of white A4 copy paper to write out the source document (right).

**Fig. 2:**
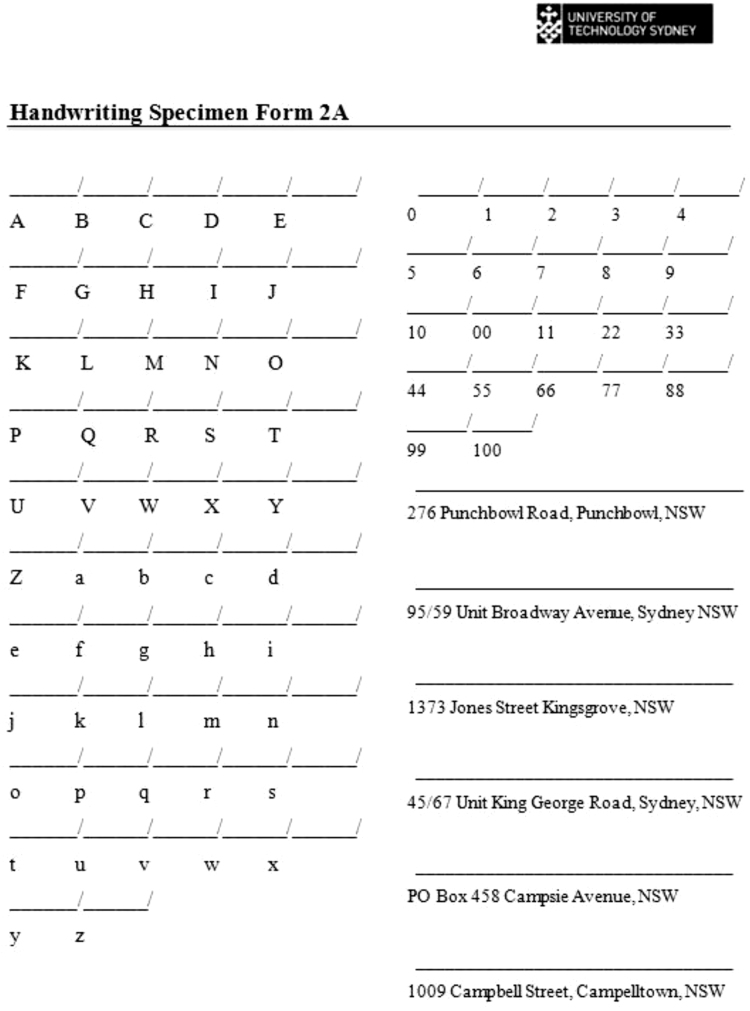
Upper and lowercase letters, numbers and postal addresses written out by participants.

**Fig. 3:**
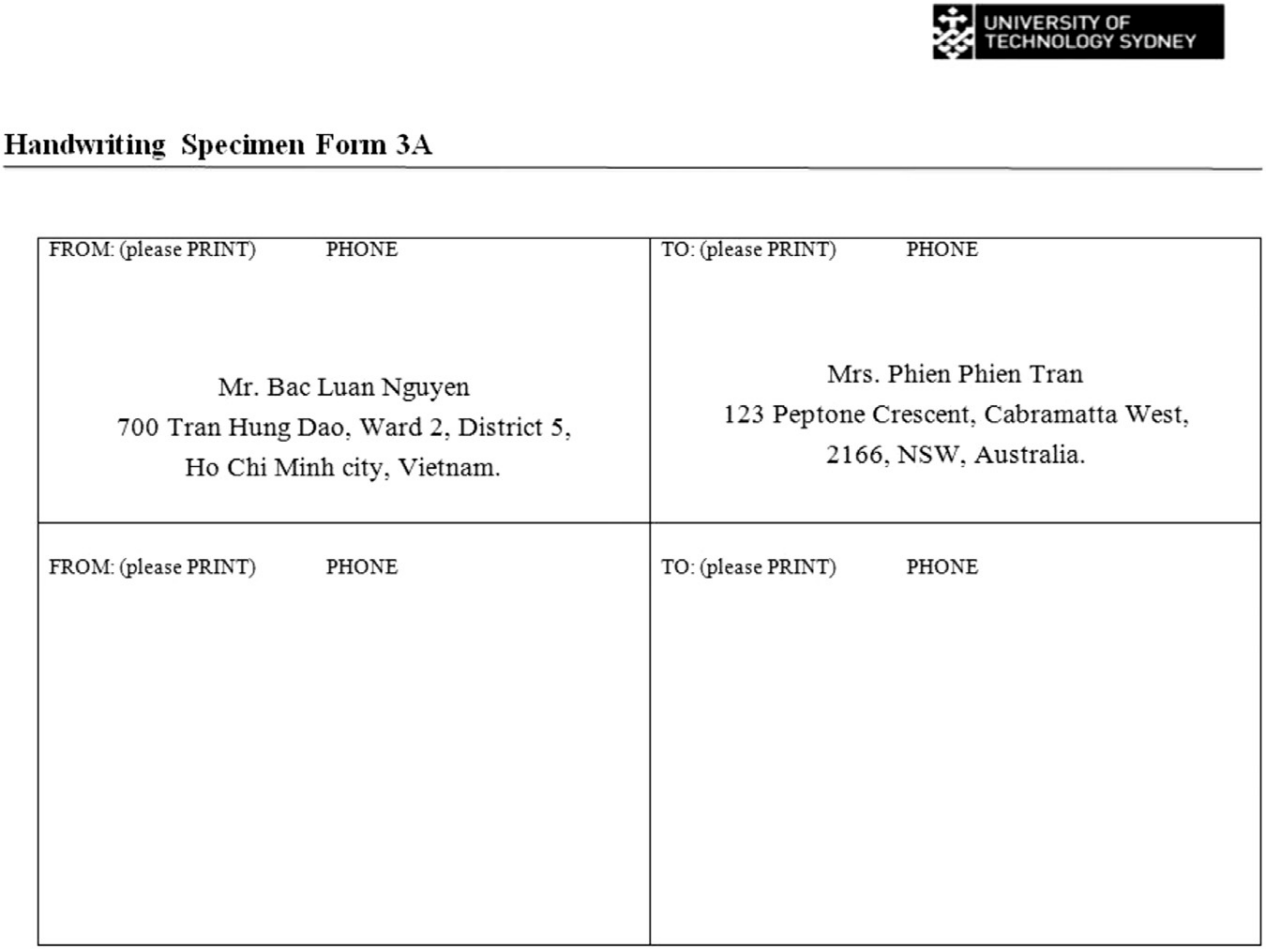
Space provided for participants to write out two postal addresses.

The method for handwriting collection is conveyed in Agius et al. [1]. Briefly, seventy-four writers completed the handwriting collection package (37 Vietnamese and 37 English Australians). The participants were required to copy out the source document (Fig. 1); the alphabet in upper and lower case and the numbers zero to ten (Fig. 2); and postal addresses (Fig. 2, Fig. 3). The source document contained all twenty-six letters of the alphabet, upper and lowercase, and the numbers 0–9. It was copied down onto a 24-lined piece of A4 white copy paper with the lines 0.8 cm apart and a 2 cm margin [1].

**Fig. 4:**
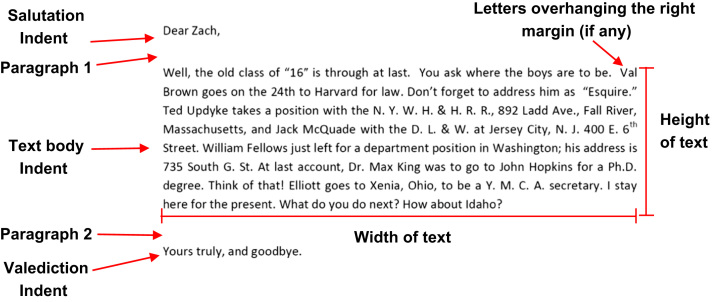
Diagram of the spatial features recorded from the source document.

**Fig. 5:**
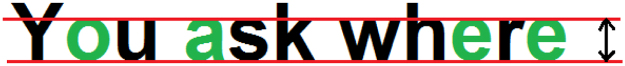
Example of measuring the average height of ‘a’, ‘e’ and ‘o’ from the text. The distance between the top and bottom lines of best fit (red lines) was measured.

The handwriting features that were chosen for extraction were separated into writer, spatial and construction characteristics. They were recorded or coded depending on the type of data, i.e. the age of the writer was recorded as is, however descriptive information like the direction of the stroke was coded zero for anticlockwise and one for clockwise. The spatial characteristics were extracted only from the source document as it had a sufficient quantity of writing as opposed to the other pages of the collection package. Fig. 4 is a diagram demonstrating the spatial characteristics extraction process. Table 2 summarises the codes of the qualitative spatial characteristics. Fig. 5 provides a visual depiction of how the feature, the average height of ‘a’, ‘e’ and ‘o’, was measured.

**Table 2:** Qualitative spatial characteristic codes.

***Spatial characteristic***	***Code***	***Code description***
Salutation indented	0	No indent
	1	Indent
Text indented	0	No indent
	1	Indent
Valediction indented	0	No indent
	1	Indent
Paragraph (1)	0	No space between salutation and text body
	1	Space between salutation and text body
Paragraph (2)	0	No space between text and valediction
	1	Space between text body and valediction

The construction characteristics for ‘g’, ‘h’ and ‘th’ were extracted from the source document and from the individual upper and lowercase sections in Fig. 2. They were not extracted from the postal addresses in Fig. 2 as the writing tended to be cramped because there was not much space to write out the postal address, or Fig. 3 as many of the participants wrote the addresses in uppercase. The characters ‘M’, ‘0’, ‘7’ and ‘9’ were taken from all three forms in the collection package. Table 3 shows how many of each character had their construction characteristics coded in the collection package for each person.

**Table 3:** The number of characters that had their construction characteristics extracted and coded.

**Character**	**Count**
g	10
h	28
th	13
M	8
0	12
7	8
9	7

Examples of the construction characteristics extracted for each character and their numerical code are presented in Table 4. This is provided in order to visually convey how the data was created, allowing researchers to make an informed selection of (a) statistical technique(s) which may be more suitable to analyse and model the data.

**Table 4:** Examples of all of the construction characteristics extracted for each character and their respective codes.

## References

[1] Agius A., Morelato M., Moret S., Chadwick S., Epple R., Jones K., Brown J., Roux C. (2018). Using handwriting to infer a writer's country of origin for forensic intelligence purposes. Forensic Sci. Int..

