# Supplementary material for: Dataset of coded handwriting features for use in statistical modelling
Source: Data Brief. 2017 Dec 13;16:1010–24. doi: 10.1016/j.dib.2017.12.014 (PMC5752089; doi:10.1016/j.dib.2017.12.014)
Supplement: Supplementary file 1 — Supplementary material [file mmc1.docx]

Declarations of interest

None.
